# From cohort to community: The emotional work of birthday cards in the Medical Research Council National Survey of Health and Development, 1946–2018

**DOI:** 10.1177/0952695121999283

**Published:** 2021-05-20

**Authors:** Hannah J. Elizabeth, Daisy Payling

**Affiliations:** London School of Hygiene and Tropical Medicine, UK; University of Essex, UK

**Keywords:** cohort study, history of emotions, history of health, kinship, social science

## Abstract

The Medical Research Council National Survey of Health and Development (NSHD) is Britain’s longest-running birth cohort study. From their birth in 1946 until the present day, its research participants, or study members, have filled out questionnaires and completed cognitive or physical examinations every few years. Among other outcomes, the findings of these studies have framed how we understand health inequalities. Throughout the decades and multiple follow-up studies, each year the study members have received a birthday card from the survey staff. Although the birthday cards were originally produced in 1962 as a method to record changes of address at a time when the adolescent study members were potentially leaving school and home, they have become more than that with time. The cards mark, and have helped create, an ongoing evolving relationship between the NSHD and the surveyed study members, eventually coming to represent a relationship between the study members themselves. This article uses the birthday cards alongside archival material from the NSHD and oral history interviews with survey staff to trace the history of the growing awareness of importance of emotion within British social science research communities over the course of the 20th and early 21st centuries. It documents changing attitudes to science’s dependence on research participants, their well-being, and the collaborative nature of scientific research. The article deploys an intertextual approach to reading these texts alongside an attention to emotional communities drawing on the work of Barbara Rosenwein.

In 1962, the week they turned 16, around 5000 teenage boys and girls received a birthday card from the Medical Research Council’s National Survey of Health and Development (NSHD). These teenagers were part of an ‘exclusive club’, their birthday cards talismans of their membership of a group that would grow up ‘a little bit special’, ‘looked after’ and looked *at* by an evolving NSHD.^1^

In 2016, 54 years later, study member David Ward extoled on the NSHD website, ‘The survey and its surviving members…are celebrating the 70th – platinum – anniversary of a relationship that has always seemed as much a fond partnership as a research project’ (Ward, 2016). The celebration of the study had by this point evolved from birthday cards to birthday parties, the cards themselves designed in-house with increased care, emotional labour, and an element of competition.

Beginning as a one-off study of maternity services, the NSHD examined the experience of all mothers who recorded births in Scotland, Wales, and England during one week of March 1946. It evolved into Britain’s longest-running cohort study, the first of five birth cohort studies that have mapped the health and development of five generations of British children.^2^ Their data has been used in over 6000 published papers and around 40 books (Pearson, 2016a: 5), shaping our understanding of health inequalities and contributing to improvements in maternity and early-years care.

After the first questionnaire given to mothers in 1946, a sample of the children were inducted into follow-up studies, which are still ongoing. Data was collected at birth (1946), in the period 1947–50 (ages 1 to 4), from 1951 to 1961 (ages 5 to 15), from 1962 to 1981 (ages 16 to 35), in 1982 (age 36), in 1989 (age 43), in 1999 (age 53), from 2006 to 2010 (ages 60 to 64), and in 2014–15 (ages 68 to 69; Wadsworth *et al.*, 2006: 50; Welshman, 2012: 177). From the initial investigation into childbirth and the financial ‘cost of pregnancy’, the aims of subsequent studies have changed. In the 1950s, investigation turned to the effects of family and environmental factors on growth and educational attainment in the wake of the 1944 Education Act. In the 1960s, the NSHD shifted to the occupational outcomes of education and early life circumstances, investigating pathways to ability and attainment, ‘problem’ behaviours, and respiratory disease. Since 1977, the studies have increasingly explored pathways to physical and cognitive ageing using data collected throughout life in conjunction with newly taken measurements, including blood pressure, lung function, and, most recently, balance and grip tests (Wadsworth *et al.*, 2006: 50).^3^

Since turning 16, study members have annually received birthday cards from the survey staff.^4^ Originally produced in 1962 as a method to record changes of address, these cards have come to represent a great deal more. No longer mere missives designed to prevent loss to follow-up (the loss of participants who were previously members of a research group), the cards mark, and have helped create, an ongoing evolving relationship between the NSHD and study members, eventually representing a relationship between the study members themselves. For some of the researchers and study members, the annual cards hold a talisman-like quality; a sign of a shared history as emotional as it is scientific. They provide a yearly snapshot of the changing visions the NSHD had of itself and its subjects, capturing a history of scientific and emotional communication. By analysing these cards, this article uncovers the growing awareness of the importance of emotion within British social science research communities from 1946 to the present, documenting changing attitudes to research participants, their well-being, and the collaborative nature of scientific research.

At their inception, the birthday cards disseminated a simplistic vision of the NSHD as a scientific study. The study members were envisioned as a studied audience with few needs beyond what little simplified scientific knowledge and gratitude would ensure future participation. As the survey continued, the divide between the NSHD and the people it surveyed dissolved as their emotional and physical well-being were increasingly foregrounded in the study’s mechanics, and as the NSHD began to feel an increased duty of care. These changes aligned with evolving attitudes towards the place of ethics in social scientific studies, the need for public engagement, and the value of interdisciplinary research combining quantitative and qualitative study (Reubi, 2012). This changed the relationship between the NSHD and its study members from surveyors and surveyed to one more of collaborative participation. As the survey’s aims developed, the cards became increasingly intertextual texts that marked the presence of an increasingly intertwined set of actors: an emotional community of researchers and an emergent textual community of participants.^5^ This article unites a history of science with a history of emotions, drawing out the emotive and relational elements of scientific research involving lay participants by examining the production context, execution, dissemination, and reception of an innovative method of science communication – the NSHD birthday card. An intertextual approach – the referential nature of texts, their dialogic quality, their ‘work-like’ aspects, and their dynamic polysemic meanings (Bakhtin, 1981; Lacapra, 1980: 250; Morris, 1992; Vargova, 2007: 415) – foregrounds the primacy of prior audience knowledge, emotion, and context in shaping the construction, reception, and meaning. By taking the NSHD’s birthday cards – and the feelings-work they enacted – seriously, we can track the researchers’ conscious community building, demonstrating how the NSHD’s institutional identity as Britain’s longest-running cohort study melded with its increasingly intimate relationship with its study members.

## From maternity study to longitudinal health survey

The technological innovation of the sample survey in epidemiology was integral to the development of 20th-century public health (Porter, 1996: 202–5). The interpretation of statistics increasingly shaped how population health was viewed by policymakers and what actions were taken to improve it (Szreter, 2002). But it also encouraged a new, more comprehensive conception of members of the public as subjects of, and participants in, the research and governance of health (Crook, 2016: 295). Increasingly it was understood that the public could contribute to the nation’s health by being the subjects of study, alongside more direct interventions such as vaccination (Mold *et al.*, 2019). Much of the literature on the development of surveys in the 19th and early 20th centuries has been influenced by Foucauldian notions of surveillance and power, describing surveys as instruments deployed by middle-class philanthropists and social reformers ‘to know, to contain, to control, and to speak about the poor’, often using terms of moral judgement (Armstrong, 1983: 51; Koven, 1991: 370). In the 20th century, social scientists assumed this mantle, conducting surveys on unemployed or working people ‘whose lives were impoverished and marginalised’ rather than the ‘prosperous and secure’ (Lawrence, 2013: 274–5). These studies, dubbed “gentlemanly” social science’ by Savage to emphasise their ‘masculine and bourgeois aspects’, relied on documentary analysis or the observations of ‘“cultivated” observers’, such as health visitors (Davies, 1988; Savage, 2010: 12, 94). Researchers from ‘within a broader gentlemanly infrastructure of elite schools, universities, [and] social organisations’ focused on mapping and classifying populations, giving little time to ‘the narratives and statements of the “researched”’ (Savage, 2010: 94–9). When they were published, the studies’ ‘language of graphs, tables and statistics defined an elite readership and excluded’ the surveyed from their audience (Koven, 1991: 370).

The 1920s and 1930s saw the development of public opinion research and representative sampling techniques. Alongside wider reportage and higher literacy rates, this enabled more people to engage with, and relate to, the data produced through surveys (Osborne and Rose, 1999: 379). In 1937, Mass Observation’s ‘anthropology of ourselves’ marked a turn towards understanding everyday life and offered an alternative model to the expert ‘observer’ (Jardine, 2018: 58). The increased importance placed on civilian contributions during World War Two made ‘understanding the politics of everyday life’ matter even more; ‘as much, perhaps more than, mapping parameters of Britain’s social problems’ (Agar, 2003: 227–8; Beers, 2006: 181, 189; Lawrence, 2013: 278). Recruited by Mass Observation or approached by the Wartime Social Survey, members of the public were asked questions directly and encouraged to speak their mind, rather than being observed and reported on by ‘expert’ informers (Summerfield, 1985: 443–6). They were participants *in* and subjects *of* research (Marsh, 2009: 215). Warmed up by the publicity given to organisations like Mass Observation, many members of the public enjoyed the process; though there were inevitably some who resented giving their time and information (Payling, 2020: 317).^6^ Nonetheless, while British government departments made more frequent use of direct-response questionnaires, Savage argues most social scientific enquiries continued in the pre-war ‘gentlemanly’ tradition.

After the war, the wealthy donors of early 20th-century social science were ‘thinner on the ground’, but private funding, funnelled through trusts, foundations, and charities, met the needs of projects like the NSHD (Savage, 2010: 101). NSHD researchers, led by James Douglas, were part of the existing elite social science network. Douglas had worked as a member of the Ministry of Information’s ‘pioneering’ Wartime Social Survey and had been influenced by social biologist Lancelot Hogben’s analysis of the factors affecting population growth. There Douglas met and befriended the London School of Economics sociologist T. H. Marshall, who introduced him to Population Investigation Committee founding member and demographer David Glass. Glass hand-picked Douglas to direct the NSHD’s original investigation into maternity services (Ramsden, 2014: 132–3).

Douglas’ plan was to map every birth in one week of March 1946, and interview the mothers about their life circumstances and birthing experiences. With 16,695 registered births across Great Britain, it was an ambitious undertaking. Drafting in help from local medical officers of health and health visitors in 424 local authorities, Douglas managed to reach 13,687 mothers (Wadsworth *et al.*, 2006: 50). Of these, 7287 answered a questionnaire about their use of maternity services, and 6400 were asked about the costs associated with their baby’s birth (Welshman, 2012: 179). Health visitors acted as ‘expert’ observers. However, in a small break from the gentlemanly tradition, the 1946 study privileged the voices of the mothers, with interviewers asked to write ‘exactly what the mother says’.^7^

Initially a one-off survey, follow-up studies were planned, with the 13,687-strong population of mothers reduced to a sample of 5362 children (Wadsworth, 2010: 132). Sampling marked a break with the gentlemanly ‘imperative’ to map whole populations, or focus solely on populations of interest, with the inclusion of people from higher socio-economic brackets narrowing the gap between researcher and researched (Bulmer, 1985: 22; Hakim, 1985: 41; Savage, 2010: 7, 99). However, ‘expert’ observers continued to be used throughout the 1940s and 1950s (Wadsworth, 2010: 126). The 1948 questionnaire prompted health visitors to ‘comment freely on the state of the dwelling,…dampness, light and ventilation…[and] the bodily care of the baby’.^8^ This adopted a moralising tone when the health visitor was asked to rate each as ‘good’, ‘fair’, or ‘poor’. Similarly, the 1950 questionnaire asked health visitors whether the mother was ‘willing or unwilling’ to accept advice, and her relationship to the child’s father, hoping ‘the answers…would separate the very good and the inefficient mothers from the large group of “average” mothers’.^9^

In the 1950s, the responsibility of observation was given to school staff. From 1952 until 1961 there were various interviews with mothers conducted by school nurses, questionnaires completed by teachers on the children’s progress, cognitive tests given to the children, and physical examinations by the school doctor. The 1957 questionnaire given to mothers required school nurses to observe whether the child in question had been vaccinated, and if the family possessed a telephone, car, or television, ‘from your own knowledge. DO NOT ask the mother directly’.^10^ Although still utilising the ‘expert’ observer, the focus of the study had started to shift away from the moral concerns of ‘good’ motherhood and family relationships, towards a preoccupation with economic and social class, though as Lawrence notes, studies of class were still imbued with a distinct morality (Lawrence, 2014: 233; Savage, 2010: 172, 187).

By the 1960s, the NSHD had moved further still from the gentlemanly tradition, with an increasingly ‘professional and “demoralised”’ focus on class, social mobility, and affluence. In 1961, the NSHD questionnaire given to school-leavers asked about career ambitions and contrasted these with those of their parents.^11^ The NSHD’s findings played a ‘particularly important role’ in the Plowden Committee of the Central Advisory Council for Education (England), which examined primary education and the transition to secondary education between 1963 and 1967 (Ramsden, 2014: 136). But the biggest shift came in the practicalities of administering the study. In 1961, study members turned 15 and completed their final year of compulsory education, leaving behind the institutional network of ‘expert’ observers who made the study possible. This impacted more than data collection, as one study member recalled:

When I was fifteen…I decided I wasn’t going to fill in the questionnaire that I’d been given by the head teacher. I said ‘No, I’m sorry, I’ve decided I’m not going to participate anymore.’ Well, he was so angry with me. He said ‘How selfish could you be? It hasn’t been about you at all, it’s about future generations’, and I never forgot that. (children90s, 2016)

Douglas feared as study members left school, with some leaving home as well, they would be lost to the NSHD. Loss to follow-up could compromise the validity of the study, with results biased by the differences between those who dropped out and those who remained, negatively affecting the representativeness of the NSHD (Dettori, 2011; Robinson *et al.*, 2007: 757). To prevent this, Douglas alighted on the idea of sending study members a birthday card, reminding them of their participation, marking the longevity of the study so far, and providing the opportunity to notify the NSHD of any change of address.

In the last week of February 1962, the first cards were sent out, bearing the message ‘The National Survey wishes all its 5,000 young people a very happy sixteenth birthday’.^12^ These cards marked a shift in responsibility for participation in the study. No longer were questionnaires aimed at mothers, or mediated through teachers and school doctors. Instead communication came directly to study members, their continued participation now entirely reliant on their continued active consent. Although the 1965 questionnaire asked the interviewer to ‘comment on any unusual aspect of this young person’s health, career or personal circumstances not clearly brought out in the interview’ in the ‘expert’ observer fashion, the 1966 questionnaire (sent at age 20) was a self-completed postal survey that included space, albeit limited, for participants to offer their own reflections. Consequently, the only information collected about the study members in 1966 was provided by themselves.^13^

Innovative at the time, birthday and Christmas cards have since been used in numerous longitudinal cohort studies. Present-day researchers are hesitant to claim cards actually prevent loss to follow-up, describing them as ‘a nice gesture’ and ‘better than nothing’ – carrying thanks as much as a reminder to participate (Brueton *et al.*, 2014; Norvell, Dettori, and Chapman, 2016). The limited compensatory nature of birthday cards –sent in return for the valuable time and data given by study members– might seem to indicate the presence of an unequivocal ‘gift relationship’ between study members and the NSHD, following Richard Titmuss’ model, wherein the individual ‘should freely give of him or herself in order to benefit the community’ (Mold, 2016: 33; Titmuss, 2018[1970]). Some NSHD study members viewed their participation in terms of benefiting future generations. But others see themselves as the beneficiaries, describing themselves as ‘lucky’ to have been ‘chosen as part of it’, receiving enduring attention from the scientific community, which later included diagnostic medical examinations.^14^

The birthday cards represent one method among many that have ensured the NSHD’s longevity. Still, as objects simultaneously public, mass produced, and personal, they offer unique insight into the NSHD’s institutional identity. In 2011, a study member described the bridge between researchers and study members the cards represented: ‘over the years I began to feel I knew the team members, although I had never met any’ (Pearson, 2011: 24). For Diana Kuh, the NSHD’s director between 2007 and 2017, this feeling is key.^15^ Although the birthday cards ‘are very popular’, researchers’ personal replies to any complaints, comments, and queries have also contributed to the ‘strong identity that the participants and the research team have with the NSHD’, which is a ‘major factor in the high response rates and the continuing success of the study’ (Kuh and Cooper, 2014: 15). Through the NSHD’s representation of itself in the birthday cards, we can glimpse how it viewed its relationship with participants, and later sought to represent its research and the enduring commitment of its study members.

## ‘Happy birthday from all of us at the National Survey of Health and Development’: Building an emotional community through birthday cards

Greetings cards are an ‘object of communication’ deployed to ‘reaffirm and re-establish relationships’ through emotive and ritualised language; they function as a rationalisation, objectification and fictionalisation of relationships by taking personal missives and turning them into ‘standardized verse’ (Papson, 1986: 100). Despite this, they remain personal, their sending an act of ‘kin-work’: emotional labour that pursues the maintenance of an emotional community by creating an ‘obligation’ among its participants to communicate and perform reciprocal emotional labour to maintain the community (di Leonardo, 1987: 442–52). Papson argues that because greetings cards demand similarly prefabricated standardised responses – the thank-you note or reciprocal card – they weaken the social relationships that might otherwise exist in interactions that transcend the textual. However, this both undervalues the kin-work involved in choosing and sending a card, and the manner by which institutions communicating with large audiences have deployed greetings cards in an attempt to create an emotional relationship, and in some cases a community, where there might not otherwise have been one.

Read cynically, in mimicking the kin-work involved in sending a birthday card, the NSHD demanded a response (in this case not a thank-you letter or card, but a change of address form or survey) and falsified intimacy between itself and its study members. Indeed, ‘the original intention of the birthday card’ was ‘tracking’, rather than the science communication, thanks, information gathering, kin-work, and tracking that became hallmarks of later cards.^16^ However, uncharitable readings dissolve when the production context is considered, revealing the manual, emotional, and intellectual effort behind them. The labour behind these cards was varied and significant, and increased as they became a part of the NSHD’s annual routine.

Over time, the cards became the collaborative creation of an emotional community of researchers attempting to build a relationship with study members who were imagined simultaneously as individuals, as vital data points fundamental to the ongoing success of the study, and later, as an emotional community. The cards came to hold a gift-like quality for the researchers who made them; given in thanks and celebration to acknowledge the fundamental importance of participants to the ongoing success and longevity of the study, but also as a material invocation of the more nebulous gift of emotional and intellectual labour the NSHD researchers bestowed on participants. As Louise Purbrick argues following Mauss, to ‘receive a gift is to accept the giver along with their offering; it is to allow the giver a part of the receiver’s future’ (Purbrick, 2014: 12). Thus, as gifts and signifiers, the cards annually worked to construct an emotional community of participants and researchers by dwelling for a moment on the shared labour that had come before, and which would continue into the future. With every celebrated birthday, the NSHD was able to reinforce its identity as an institution built on enduring work, emotional and scientific; its very longevity ensured by the pride it instilled through annual celebrations of its ever increasing history.^17^

Here it becomes necessary to offer some definitions. Throughout this article we use the terms ‘emotional community’ and ‘textual community’ to describe the NSHD’s researchers and study members, drawing on the work of Barbara Rosenwein and Brian Stock. Rosenwein defines emotional communities as ‘social communities’ whose shared ‘systems of feeling’ are defined by what ‘these communities (and the individuals within them) define and assess as valuable or harmful to them’ (Rosenwein, 2002: 842). These communities can be recognised where ‘people adhere to the same norms of emotional expression and value – or devalue’ and are created when people are ‘animated by common or similar interests, values, and emotional styles and valuations’ through face-to-face contact or shared texts (Lynch, 2016: 4; Rosenwein, 2002: 842; 2006). This definition applies to the scientists working at the NSHD, but the study members themselves are trickier to recognise. Face-to-face interactions between participants were largely absent until the 65th birthday party celebration, making shared ideologies harder to track. Theirs was a ‘textual community’ under annual construction.

Stock uses the term *textual community* to describe the social circles formed by sharing texts, and the consequent shared knowledge, expectations, and rules that are imparted by texts. Since birth, the study members have shared certain texts and experiences: they have been surveyed 24 times, interacting with the same birthday cards, questionnaires, and booklets while simultaneously undergoing identical medical examinations and interviews. Later, when it became clear the study would persist, they also lived with the shared knowledge of more tests and texts to come. Study members grew up knowing they were one among many, each recipient made aware of the numerous others also in receipt of the cards, annually reminding them of their obligation to the NSHD and to one another; each card marking another anniversary in this relationship. The cards themselves were consciously intertextual (see Figures 3 [Fig fig4-0952695121999283] [Fig fig5-0952695121999283] [Fig fig6-0952695121999283] [Fig fig7-0952695121999283] [Fig fig8-0952695121999283] and 9). Referencing both previous publications and research yet to come, they represented a lifetime structured by NSHD interventions. The bonds of this textual community strengthened as more NSHD texts were added to its canon, a sense of shared authorship in the NSHD’s success and publications created through each exchange of thanks and data.

With this degree of shared history and shared future it is tempting to now see an emotional community in the cohort constructed and surveyed by the NSHD interventions. But when did study members become an emotional community and when did the cards transform from mere information requests to something more personal and affective? The repeated testing and giving of testimony seems akin to a construction of ‘shared vocabularies and ways of thinking’ resultant in ‘a disciplining function’ (Rosenwein, 2006: 25). Certainly the health and lives of the study members were changed by growing up under the NSHD’s peculiar surveillance – what is known scientifically as the Hawthorn Effect (McCambridge, Witton, and Elbourne, 2014). Study members were asked repeatedly to consider their future when children and teenagers; to reflect on their past as adults. While the exact normative effects of this solicited self-reflexivity are hard to unpick, the health effects of the study are not. NSHD study members are, if not healthier than their unsurveyed peers, more informed about their health. The comprehensive and frequent tests required by the study have allowed early diagnosis and intervention to become a norm for its participants (Pearson, 2011).^18^

## ‘A huge bore’: Producing and sending the first cards

Douglas thought that birthday cards were ‘perfect; one week…we could just blast them off’. However, knowing names, addresses, and vague birthdays did not make sending the cards a simple task. Brightly illustrated, the first cards were designed out of house for no fee at the behest of Rachel Douglas – Douglas’ wife – through her publishing company, borrowing the labour of her book jacket designers. Even then, physically sending ‘all those damn things’ to the right address with accompanying paraphernalia ‘was really a huge bore’. Without the routine and experience that would later develop, these first cards required an initial burst of intellectual and physical labour – ‘a hell of a job!’.^19^

**Figure 1.**
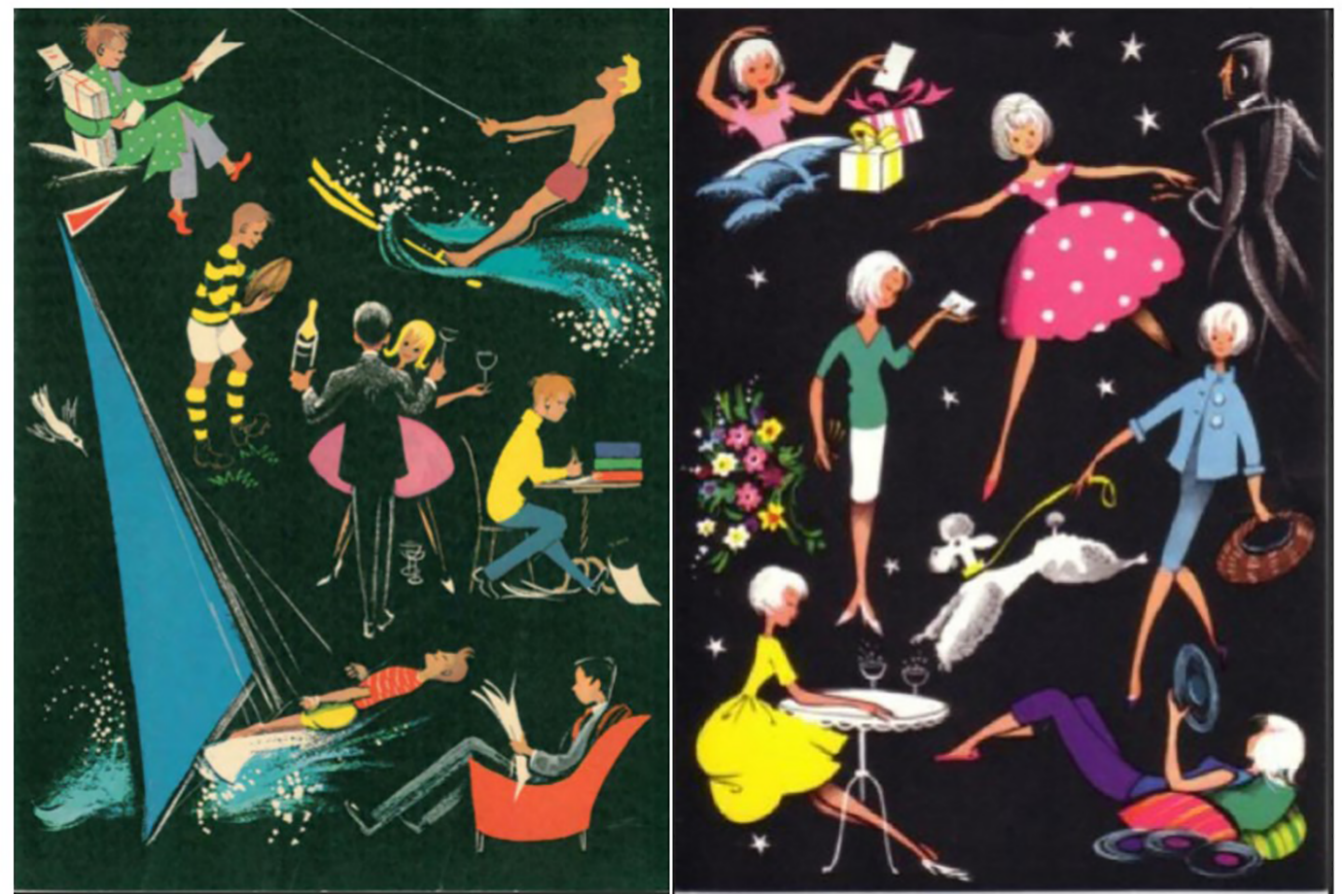
The first birthday cards, 1962.

The first card was the only card with two designs, split along gendered lines (Figure 1). While the boys’ (left in Figure 1) and girls’ (right in Figure 1) cards differed, both showed young adults engaged in various recreational activities. They had an aspirational quality, speaking to an emerging affluent consumer culture, but requiring more money and maturity than the 16-year-old recipients would likely have (Marwick, 2011: 74). For example, drinking and dancing featured in both cards, with the youths dressed stylishly. The boys’ card also featured youths engaging in exciting but expensive sports; water skiing and wind surfing. Even with the wage increases experienced by working-class teenagers in the late 1950s, these activities would likely have remained beyond the reach of many study members (Mills, 2016; Osgerby, 1992). This fanciful quality is absent from later cards, the sensitivity to study members’ emotions that later developed not yet present in the depiction of a future that many would not have access to. This aspirational tone and splitting of the audience along gendered lines was echoed in some of the surveys that preceded these cards. In line with social science’s broader interest in social mobility, participants were asked about their employment aspirations, with prompts divided by gender. Jobs carrying the most prestige or requiring the most physical exertion were suggested only to male participants: for example, boys were offered ‘Architect’, ‘Explorer’, and ‘Bricklayer’, while girls chose from ‘Art Teacher’, ‘Horticulturalist’, and ‘Chocolate Packer’.^20^

**Figure 2.**
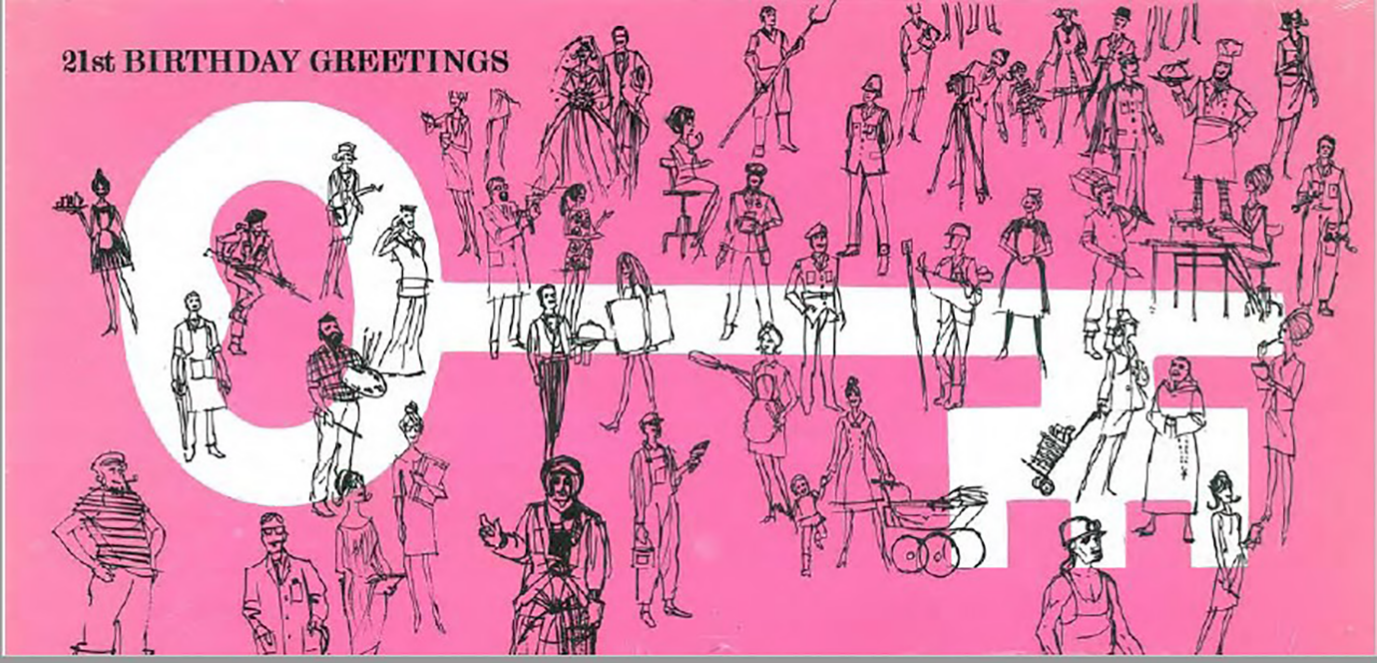
21st birthday card, 1967.

In 1967, the study members turned 21. Their NSHD card reflected this simply: it was a celebratory bright pink with a white key in the centre (see Figure 2). The key marked a traditional gift at 21, representing the opening of new doors at the age of majority. The card also reflected the work of the study up to this point. Adorning the key were small black drawings of people performing the different occupations reported or imagined by the study members in previous surveys. From age 15, study members were asked to imagine their futures through their aspirations for different kinds of work, and subsequent questionnaires asked about those occupations in detail. The 1966 and 1968 questionnaires asked about employment, current and past, and the 1966 questionnaire included a separate section for ‘final year students’, which asked them to ‘picture the job you have got or think you will actually get’ and rate it from 1 to 7 in terms of whether it was ‘a respected profession’ or a ‘rather despised profession’, and if it offered ‘great freedom to plan your own work’ or required them to ‘stick rigidly to a given syllabus’. The 1968 questionnaire asked all study members, ‘If you had your time again, what age would you choose to leave school?’.^21^ Not only did it ask study members to self-report, but it asked them to begin a process of self-reflection that would continue throughout the NSHD. Findings from these studies were written up in the NSHD’s 1968 book *All Our Future*, which concerned the so-called ‘waste of talent’ caused by social inequalities, noting that despite the expansion of higher education following the 1963 Robbins Report, able but less advantaged study members were less likely to continue their studies (Douglas, 1968). In line with the ‘socio-biographical’ turn in early 1960s sociology, *All Our Future* highlighted the way ‘individuals found their ways through social structures’ (Rustin, 2000: 44–5). This interest in the study members’ individual lives was reflected in the NSHD’s cards and questionnaires.

In the 21st birthday card, the NSHD began to address its study members as adults and as individuals within a community. Initially the NSHD had been anxious about changing the cohort’s behaviour and potentially biasing the study’s results by providing study members with too much information, lest they upset the Medical Research Council (MRC), the core funder from 1962.^22^ By the mid 1960s, however, their findings were gaining attention through frequent newspaper reports and their use as evidence in the Plowden Committee (Ramsden, 2014: 136). This shift is reflected in the 21st birthday card, which provided study members with a new level of detail about findings and went further in its attempts to build a textual relationship. It described how the information study members had provided regarding education and employment told ‘a story that is typical of the whole country’. But it also confided there were members among them ‘in more unusual occupations’, including ‘a monk, a friar, a well-known pop-singer…a bank messenger to Buckingham Palace and a girl in a casino; we don’t think that we have a Bunny Girl among our members’.^23^ By providing this information about noteworthy individuals, the card recognised that study members had individual lives but were brought together by the NSHD; articulating a form of community. Through the slightly risqué joke about ‘Bunny Girls’, the cohort, and its researchers, were allowed a touch of the permissive glamour of ‘Swinging London’, an acknowledgement from the NSHD that they were now dealing with adults, echoed in the shift from interviews to self-completion in 1966 (Mort, 2010: 350–1). This joke, alongside others, demonstrates the NSHD was experimenting with humour to build bonds with their maturing study members. Concurrently, study members were invited in the 1967 card, and in questionnaires from 1966 until 1971, to send their ‘own remarks’ and ‘add comments’ if ‘anything important happened…in the last year that we haven’t asked about’.^24^ Provided with a blank page and with reassurance that researchers would be ‘delighted’ to read comments and would ‘treat them as absolutely confidential’, study members were encouraged to write freely about their lives, adding to the biographical material collected on them as individuals and potentially shaping future research directions. The insistence on confidentiality echoed the language employed by mass-market magazines to build communities of readers through problem pages, placing the study within a much wider textual landscape (Tinkler, 1995: 8; White, 1970: 299–300). The card allowed the NSHD to position its researchers as confidants willing to hear individuals’ reflections, while explaining that it wanted the study to continue and so required the ‘constant cooperation of you all’, with reassurance given that the NSHD researchers ‘value the help you have given us through the years very highly’.^25^

**Figure 3.**
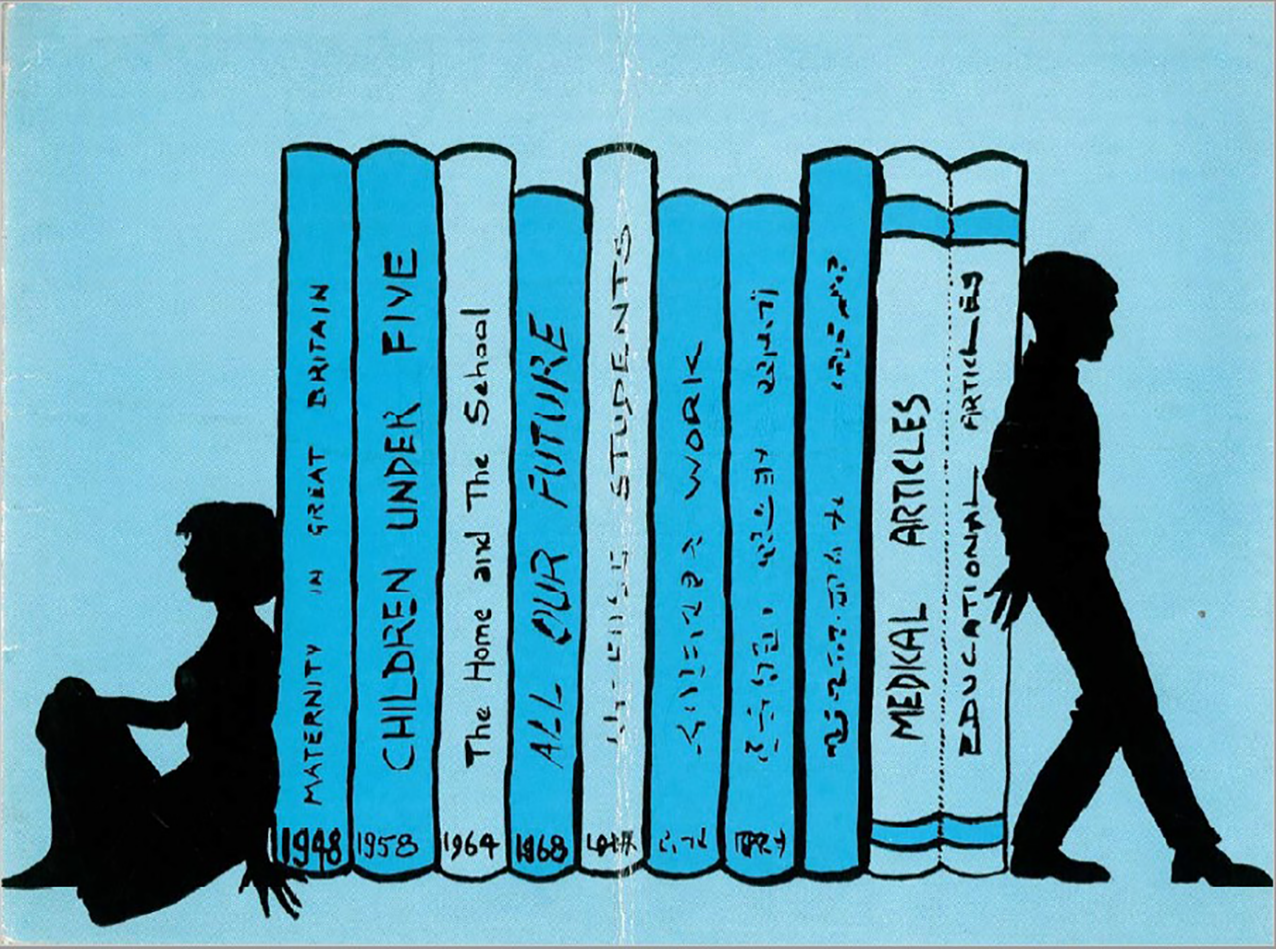
22nd birthday card, 1968.

The 22nd birthday card (1968) struck a more sombre tone (Figure 3). It was light blue, with anonymous, black silhouettes of a young woman and man bookending a row of NSHD publications depicted in a darker blue with black writing. Both figures were looking away from the publications, but each had a hand touching the books signifying their ownership of the study. The NSHD books, holding centre stage, were listed in publication order: *Maternity in Great Britain* (1948), *Children Under Five* (1958), *The Home and the School* (1964), and *All Our Future* (1968). Again, this card pointed towards the future – after *All Our Future*, six more books were imagined on the bookshelf. Two were illegible, but two indicated the NSHD would publish on ‘students’ and ‘work’. The final two were labelled ‘Medical Articles’ and ‘Educational Articles’, with ‘Medical’ clearer and larger, as if to reassure the MRC that the study had a continued focus on health alongside education and social mobility. Michael Wadsworth, who joined the NSHD as a researcher in 1968, remembers a sense that the MRC was saying to Douglas, ‘What the hell are you doing publishing all this stuff on education?’.^26^ These concerns notwithstanding, this card was as much about celebrating the publication of *All Our Future* and articulating the NSHD’s institutional identity as it was celebrating the study members’ birthdays. Inside, it read, ‘This year we are sending you a questionnaire with your birthday card at the completion of your twenty-second year, and yet another book has been written about you, called “All Our Future”, which is what you are’. It went on to express ‘delight’ in study members’ interest its publications: ‘We feel that you now realise the value of it all and we would like to thank you again for all your help and enthusiasm’. The emphasis on ‘now’ reiterated the new and more direct relationship between adult study members and the NSHD emerging from the one mediated by parents and teachers. The researchers once again encouraged study members to contact them, and to shape the study and their birthday cards: ‘If any of you would like to give us your ideas on any subject whatsoever on the last page we shall be very pleased to have them…and also to have suggestions of what you would like to hear about yourselves, as a group’, in the next birthday card.^27^

**Figure 4. fig4-0952695121999283:**
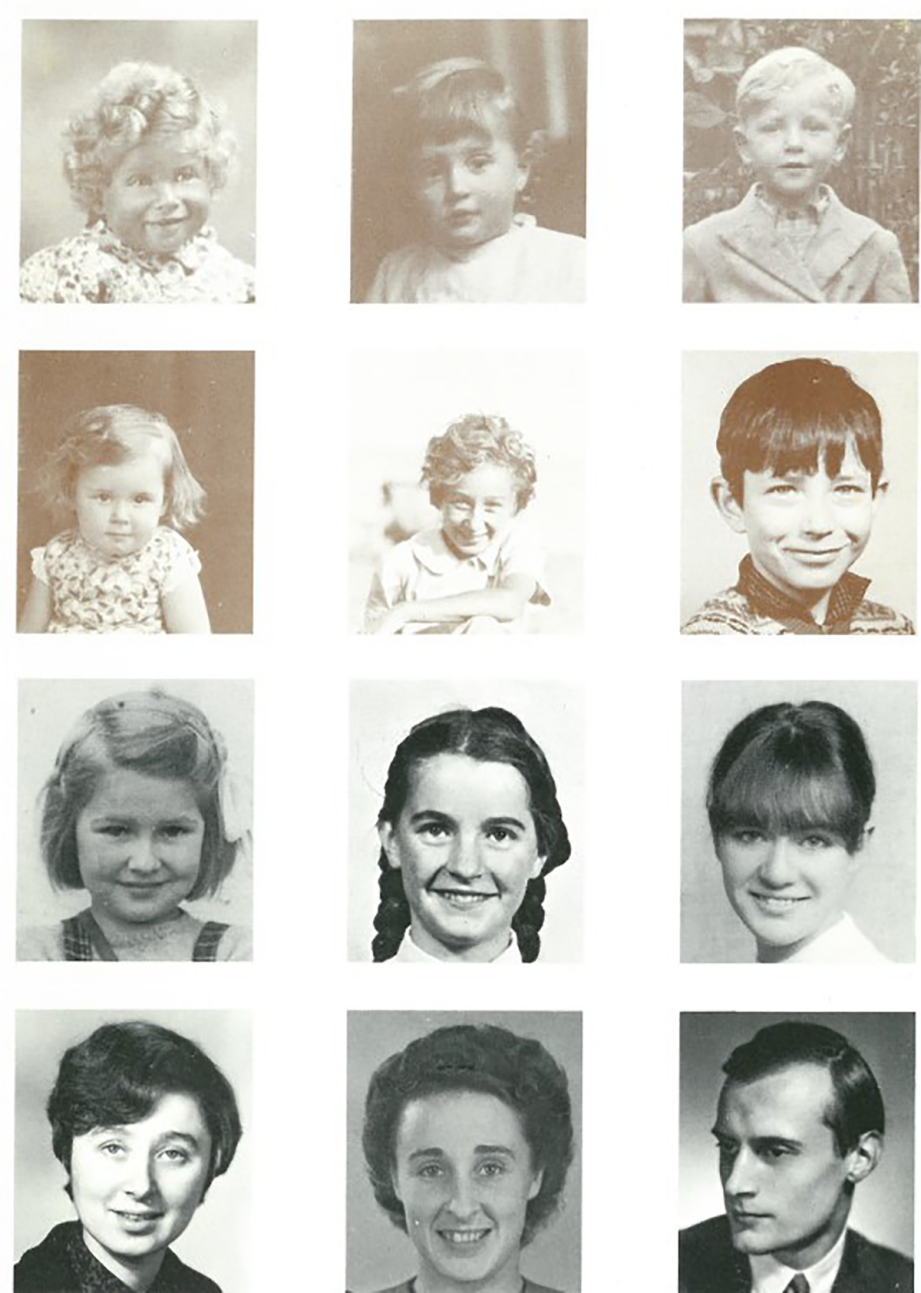
26th birthday card, 1972.

**Figure 5. fig5-0952695121999283:**
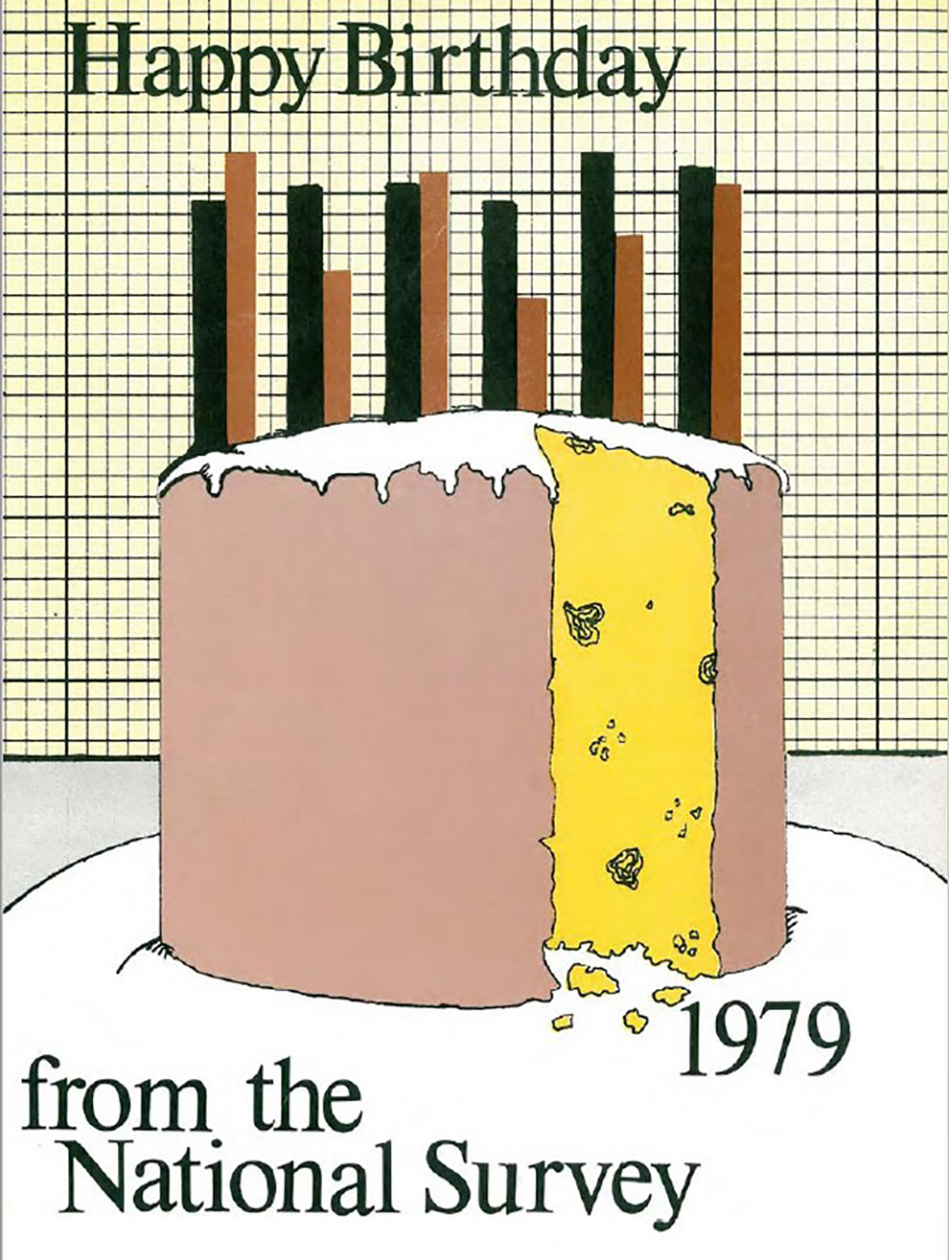
33rd birthday card, 1979.

Rather than highlighting the connection between researcher and study member, the 1979 card celebrated the NSHD’s scientific purpose (Figure 5). It depicted a birthday cake against a backdrop of graph paper. The cake’s ‘candles’ were bars on a graph, reiterating the significance of the data collected. This reflects a repositioning of the NSHD’s institutional identity once again. With continued funding from the MRC in 1977, the study turned away from the occupational and earning outcomes of education, and began tracing the pathways of cognitive and physical ageing, investigating self-care, and exploring receptivity to health promotion material (Wadsworth *et al.*, 2006: 50). In line with Rustin’s contention that ‘the history of biographical methods in the social sciences seem to have been one of fits and starts’, it also marked a shift in interest away from soliciting the reflective writing of individual study members (Rustin, 2000: 41). Absent from the 1977 questionnaire were the past- or future-oriented questions that had characterised questionnaires in the 1960s and early 1970s.^28^ Notably, the years 1978–9 were a time of uncertainty for the NSHD. Douglas was due to retire in 1979, and the MRC expected the NSHD’s research unit to fold when he left (Pearson, 2016a: 113). Wadsworth remembers the ‘awfulness of wondering what our future would be’.^29^ With the risk of closure, we can read the 1979 card’s emphasis on data as a claim to continued scientific relevance.

In July 1979, two weeks before its lease ran out in London, the MRC rang Wadsworth, who had by then taken over the NHSD’s day-to-day running. The NSHD would continue, but would do so from the epidemiology department at the University of Bristol (Pearson, 2016a: 119). Five more years of funding secured, Wadsworth celebrated by sending a 34th birthday card featuring Bristol’s famous Clifton Suspension Bridge in 1980. From Bristol, Wadsworth began work on a study of health and nutrition ‘with a level of detail that had never previously been attempted’ (ibid.: 120). This required a 46-page questionnaire and an accompanying medical examination.^30^ Carefully trained nurses visited study members in their homes to measure height, weight, blood pressure, and lung capacity. Details about members’ physical and mental health were also recorded. Members were also asked to keep a food diary for a week, a method that had provoked particular anxieties among other British epidemiological survey participants, as seen in correspondences to the 1969 Whitehall Study, where men wrote in offering many caveats alongside diet recordings (Mold *et al.*, 2019: 83–4; Pearson, 2016a: 121).^31^ The detail required by the 1982 questionnaire engendered a renewed emphasis on loss to follow-up. In a 1992 paper, Wadsworth and his team discussed the ‘steep rise in refusals’ attributed to these ‘changes in study methods’, including the ‘increased concentration of medical topics’ and the ‘first adult measures of height and weight’ (Wadsworth *et al.*, 1992: 303). These conclusions reflect Wadsworth and his team’s growing recognition, as the 1980s progressed, that the NSHD’s scrutiny was increasingly onerous. Consequently, the time and effort the survey asked of study members required a matched effort from the NSHD. In part, this recognition was a result of the slowly developing relationship between the NSHD and study members. The agency of participants was brought home by reactions to the birthday cards, responses to surveys and tests, and, in some cases, refusal to participate. The NSHD’s suspicion that study members experienced observation as burdensome was confirmed in 2010, when, during several qualitative interviews, participants reflected on feeling an increased ‘duty’ to the survey, compelled to ‘always fill in that form, even that awful food diary’, which one study member described simply as ‘nightmare. Nightmare’.^32^

Simultaneously, bioethics, which had had been developing as discipline in the US throughout the 1960s and 1970s, began to exert more influence on British science in the 1980s (Chadwick and Wilson, 2018: 186; Reubi, 2012: 350–1). As David Reubi explains, much bioethical thought centred on informed consent, and on presupposing that a research participant was a ‘person’ who was ‘able to think, act and communicate’ and decide what to do with their body, as opposed to a ‘citizen who, in the name of society and the common good, was expected to give his or her body as material for experimentation whenever physicians deemed it necessary’ (Reubi, 2012: 349, 356). These ideas found purchase through public debates about embryo research and abortion, but also because they chimed with the new political rhetoric of individual choice and responsibility popularised under Thatcherism (Chadwick and Wilson, 2018: 192; The Science and Technology Subgroup, 1991). This included the development of stricter ethical guidelines. In 1985, the NSHD was required to apply for ethical approval for the first time, submitting paperwork ‘to every ethical committee in the country’. Wadsworth remembers that the importance of ethics emerged ‘quite suddenly’. Up until 1985, NSHD researchers had ‘done whatever the hell we liked really.…There was no ethical stuff in the social sciences’. Diana Kuh, who joined the study as a researcher in 1987, recalls that consent was key: it was ‘all part of this wider thing about letting study members, and the general public, know much more about what you’re doing’.^33^ Conversely, at the start of the NSHD in 1946, ‘if someone was willing to see you, that was consent’. For the most part, ‘people didn’t think they could choose not to participate’. This was the certainly the case for some participants in the government’s ongoing Wartime Survey of Sickness in 1951, who complained afterwards that they had felt pressured to cooperate. Refusal rates were generally low in studies in the immediate post-war period, but as Mold *et al.* (2019: 76, 80) suggest, people found other ways to resist. By the mid 1980s, the NSHD was learning to build a more carefully articulated consensual relationship with its study members (Pearson, 2011: 24). The birthday cards played a role in this more consensual community. Indeed, in 1982 it was suggested that study members who had emigrated in the 1960s and left the study had subsequently chosen to rejoin when they returned to Britain, in part because they had stayed in touch through the cards (‘Untitled NSHD Clipping’, 1982).^34^

**Figure 6. fig6-0952695121999283:**
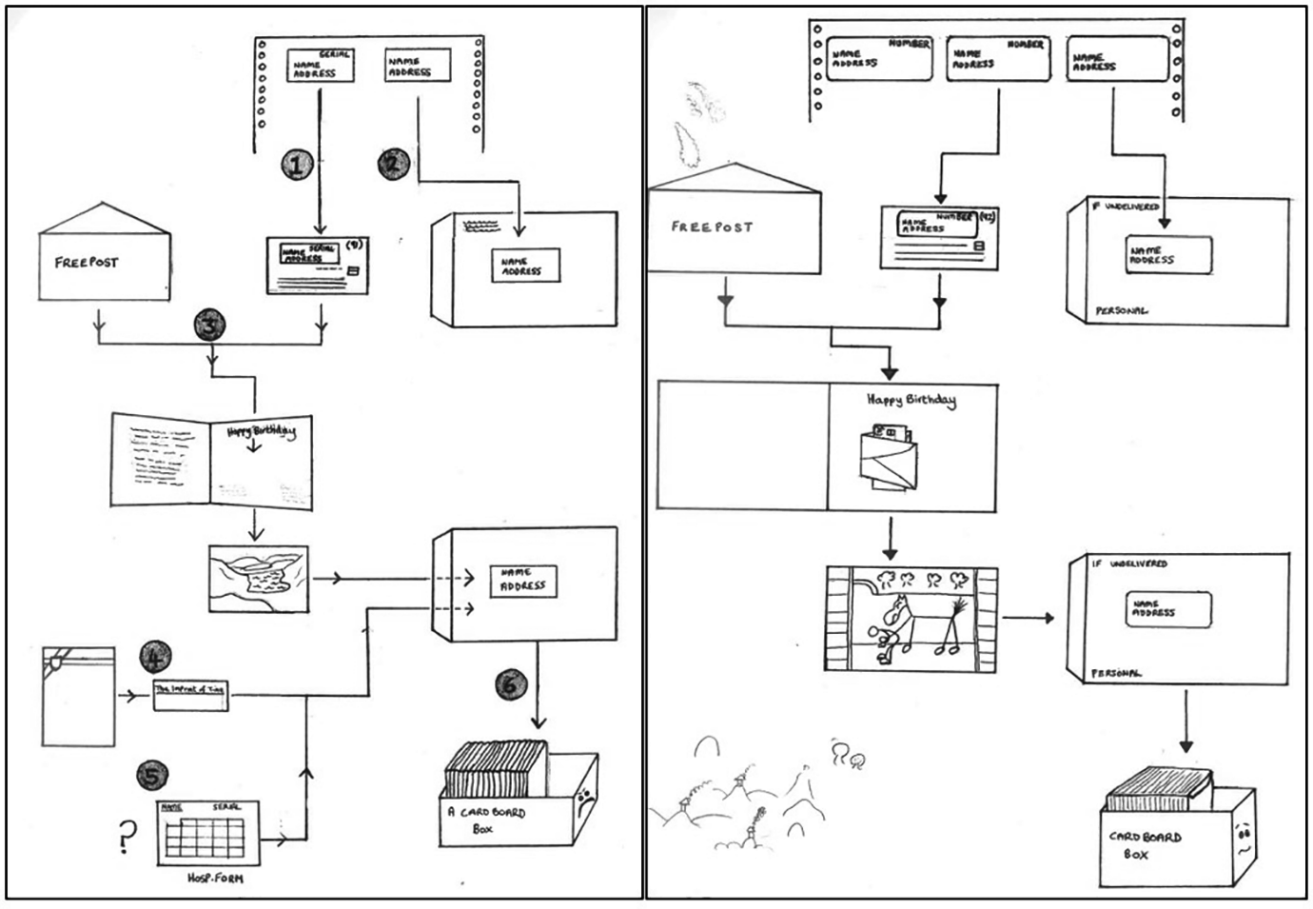
1991 card-packing flow chart; 1992 card-packing flow chart.

With more intrusive study methods and emerging ethical concerns, the birthday card took on a new significance in the 1980s – still acting as a thank you for previous participation, it now also operated as a conscious request for consent to future participation. Yet with Rachel Douglas joining her husband in retirement, the study had run out of people ‘who could do designs’.^35^ After a particularly ‘wobbly’ hand-drawn card in 1984, Wadsworth came up with the idea of a researcher photo competition. Each year researchers would submit photographs they had taken on holiday and the team would choose one for the birthday card – usually ‘a nice photograph of flowers, or…a British view’, but also, for cost and ease, ‘an image without copyright’. Kuh highlights that ‘it was such a big deal to get your photograph accepted’, and that the choosing of a winning photo becoming an important and exciting ritual for researchers. This excitement aside, producing and sending the card remained hard work. Once the design was chosen and printed, the NSHD research team ‘assembled it all’, labour that was far from simple in procedure or meaning (Figure 6).^36^

It became a ritual. Every February the NSHD research staff would ‘sort of roll up [their] sleeves’, packing the cards with an emphasis on speed and the avoidance of mistakes.^37^ For Kuh, this kind of work has an emotional value, offering ‘a way of getting the youngsters [early career researchers] realising that these are real people’.^38^ In the 1980s, biomedical research had come under fire for neglecting the ‘lived experience of health-focused encounters’ (Rickard, 2004: 165). Rickard notes that this led to an emphasis on biographical methods as researchers tried to ‘humanise medicine’ and centre patient voices. For the NSHD, whose research was increasing preoccupied with measuring and mapping study members’ physical responses, the cards and their production took the place of more creative research methods, reminding researchers their prized cohort was made up of individuals whose continuing consent was integral to the survival of the study.

Despite, or perhaps because of, the photo competition, from 1985 onwards the birthday cards were largely representations of pastoral scenes, lending them the deceptive appearance of unremarkable greetings cards. For example, the 1989 card depicted poppies in a meadow, while inside the words provided important details on the upcoming year’s work. Arriving before 1989’s weighty 51-page survey, it explained the study was investigating ‘height and rates of growth to see how far we can disentangle genetic and environmental influences’ and thanked study members profusely: ‘Of course, none of our work could have taken place without your cooperation over the years, and we are, as always, very grateful to you’.^39^ By the end of the 1980s, the cards represented a new bioethical relationship. Study members were kept informed and, through the creativity of card design and the grunt work of card production, the research team were reminded annually that study members were human beings as well as data points.

## A British view

**Figure 7. fig7-0952695121999283:**
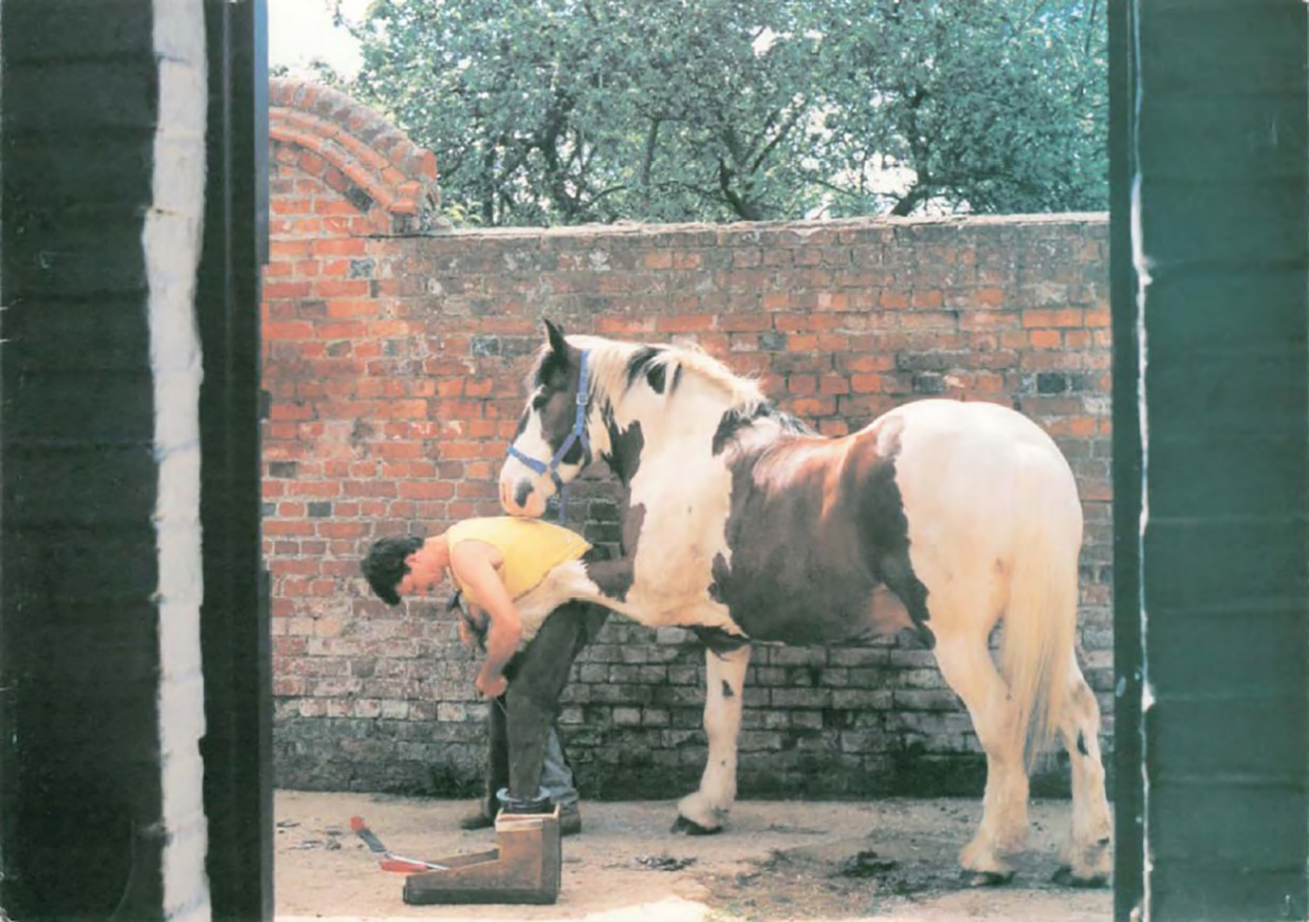
1992 birthday card: Kuh’s winning controversial holiday snap.

One way the cards’ production reflected this new relationship was through increased scrutiny over what could be used as an image. Submissions to the photo competition would not win if they failed to adhere to ‘certain criteria…like it couldn’t be a foreign view, and…horses and sunsets were out’.^40^ These unwritten rules developed in direct response to complaints about previous cards. All complaints received a reply – as did any letter the NSHD received from study members – but their content was also disseminated to researchers (Pearson, 2011: 20–4). Complaints were used to sketch the audience the cards were being prepared for, in hopes of anticipating their responses. Moreover, complaints were deployed as a warning to take the emotional needs of study members seriously, to recognise that their emotional community was different from that of the researchers. For example, the 1992 card (Figure 7), featuring a horse being shod, received complaints about being ‘rude’.^41^ While this response from participants was met with amusement by the researchers (and some disappointed exasperation from Kuh, whose winning photograph it had been), the anecdote was used to illustrate a difference of humour between the NSHD and the community it studied.

Making a joke of study members’ complaints softened the blow of getting it wrong and warned new members of the card-making team to get it right, but, more importantly, reinforced ‘a feeling of solidarity’ among researchers as an emotional community by identifying ‘differences between “us” and “them”’ (Kaufman and Blakely, 1980: 24–6). Humour is a powerful clarifier and maintainer of boundaries. Gentle though the researchers’ giggles were, they implicitly indicate the persistence of an ingroup and an outgroup (Bing, 2004: 24–6). This acknowledgement of difference through humour indicates a marked shift: the risqué jokes about Bunny Girls that had assumed a certain shared taste for ribald humour were replaced by a careful adherence to some study members’ more conservative values.^42^

Successful though the photo competition was in creating a connection between researchers and participants, it did not mark an end to design efforts. Complaints led to a redesign so cards became double-sided, giving study members the option to display the picture they liked best on their mantelpieces, while allowing participants to disguise their age, which occasionally featured on one side of cards.^43^ Around the same time, postal questionnaires once again began asking study members if they ‘would like to give further details to any questions or make any comments about the questionnaire’, but this was couched in terms of providing feedback on the data-collection process rather than participants taking the NSHD into their confidence.^44^ Cards that marked special dates or coincided with surveys or medical examinations – from 1999, grip-strength tests, blood samples, and mouth swabs became routine – warranted special design efforts. For instance, as Kuh explained, ‘We felt we needed a special card because we were going to have the 65th birthday parties’ (Figure 7). The positive reception for both card and birthday party led researchers to reintroduce designed images alongside photographs from this point. For example, the 66th birthday card featured a collage of photos featuring the number 66, combining photos with a more designed aesthetic. Mention of the labour behind these decisions, the seriousness with which study members’ opinions were treated, and the design of the cards more generally was included in the 2012 newsletter, which accompanied the 66th birthday card:

This year, two members of the team created a special 66th birthday card which we hope will be appreciated – but we have added a conventional flower photograph on the reverse, just in case there are any study members who prefer not to advertise their age! Please do let us know if you prefer one style or the other.^45^

Similarly, the 70th birthday card combined designs found in the NSHD card archive with new designs, and marked a birthday party year. The 71st birthday card featured a pastoral holiday snap on one side and bright cartoon of a birthday cake on the other. Even where two choices were made available, the polysemic nature of the more designed images led to some difficulties. As Kuh explained, the original design for the 69th birthday card prominently featured ‘all these pictures of people in Olympic poses’, which, in combination with the number, ‘had really sexual connotations.…And I actually had to veto it because I knew that there would be part of the sample that would find it quite…inappropriate’.^46^

**Figure 8. fig8-0952695121999283:**
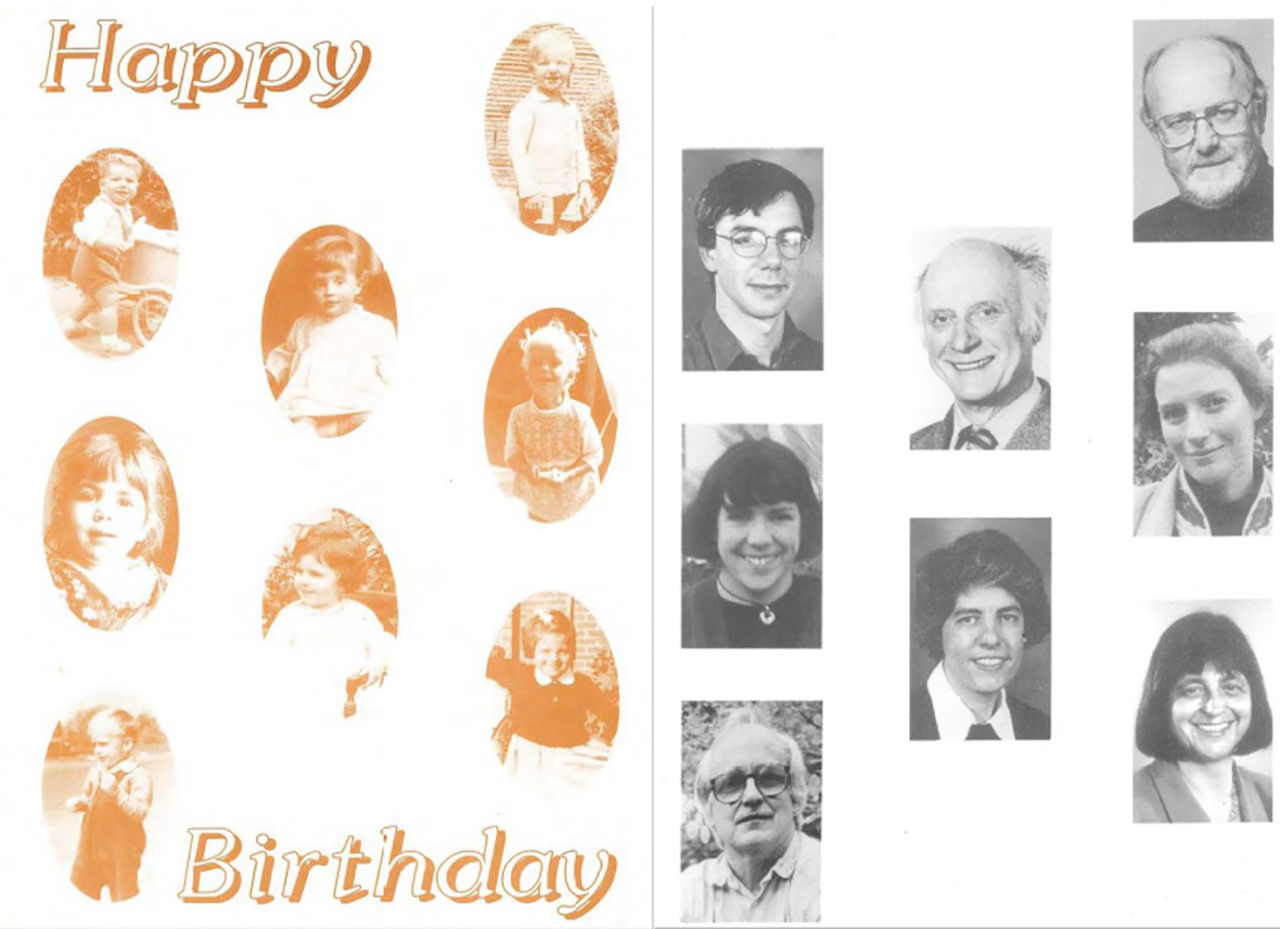
50th birthday card, 1996.

Though we can draw a great deal of insight from vetoed designs and complaints, it is important to emphasise those moments where the researchers and study members agreed. Asked what their favourite cards were, Kuh and Wadsworth pointed to those that displayed NSHD team members (Figure 8). A card featuring photographs of the researchers ‘from birth to 25 years’ was used for the first time in 1971 for their 26th birthday (Figure 4). This was consciously repeated in 1997 for their 50th birthday, but this time with the researchers displayed as adults and children. For the researchers, it was a chance to offer something of themselves to the study members, a personal addition to go alongside researchers’ signatures. The response from study members to the portraits was positive: ‘They liked that, they really liked that – it was sign of us being willing to be seen as people’.^47^

Inside, the text reminded study members of both the longevity of the study and the duration of the kin-work the birthday cards represented, explaining that the earlier card ‘put photographs of members of the study team from birth to 25 years. This time they are of current team members, and of James Douglas, who began the study’.^48^ By referencing Douglas – the man most study members had never met but whose intervention had shaped their lives – the NSHD invoked the history of the study and the years of participation already recorded. In its second iteration after 1981, Mass Observation did something similar, using the antiquated language of ‘Directive’ and ‘Mass Observation’ to invoke the organisation’s heritage and reassure participants that they were part of something long-running and larger than themselves (Sheridan, Street, and Bloome, 2000: 74–5).

**Figure 9.**
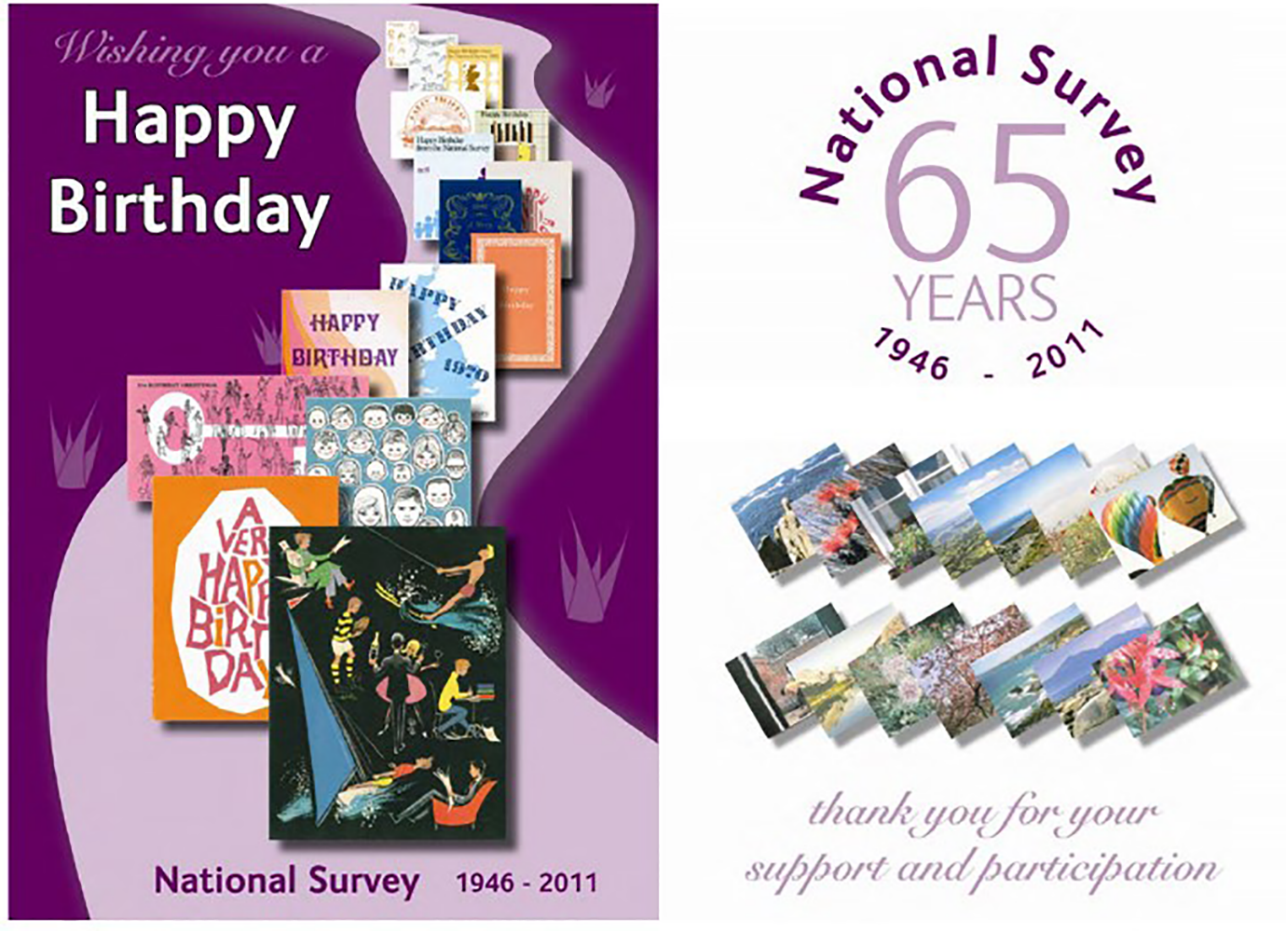
65th birthday card, 2011.

As briefly indicated above, the 65th birthday card marked a turn back to more complex designs rather than holiday snaps. Featuring some distinctive cards trailing up a road into the distance, it offered a reflection on previous designs, and on the time passed and efforts made by study members and the NSHD (Figure 7). While all the cards relied on a degree of intertextuality to remind study members of their past and future commitment to the NSHD, the 65th birthday card attempted to evoke an emotional response by deploying the prior kin-work performed by each featured card. It also functioned as invitations to a 65th birthday party celebrating the study members and the survey, ‘a thank you for the clinic visit’, and an attempt at a new loss to follow-up prevention method. Just as the cards were developed as the study members left school, the party coincided with another shift in life stage: retirement. Birthday parties had previously been viewed as impossible. At ‘60 we didn’t have the money and we were worried about damaging the sample’. Early suggestions that a birthday party might be held for study members to mark the 50th or 60th year of the study were rejected by Wadsworth, ‘in case the get-together ended up influencing participants’ life course in some way’, or, as Wadsworth bluntly put it: ‘Basically, we thought people might leave their partners and get off with someone in the study’ (Pearson, 2011: 24). Wadsworth’s concerns might be tongue-in-cheek, but they represent an acknowledgement by the NSHD that by virtue of researching the study members, they had created a community, albeit a disparate and varied one, which year on year had more in common. Wadsworth’s fears indicate a perception of the study members as an emotional community that was merely yet to meet. For some study members who attended the 65th and 70th birthday parties, this was exactly the experience they encountered. As journalist and study member David Ward recalled of the 65th party he attended, ‘The atmosphere in the room was one of rare wonderment; here was a bunch of people who did not know each other but all shared membership of a very special club to which they were very loyal. No introductions were necessary and conversation flourished (Ward, 2016).’ The need for a special card at 65 was compounded by several factors beyond the birthday parties they advertised. Between their 60th and 64th birthdays, study members had been invited to undergo a series of more intrusive tests focused on ageing as the study took on a new remit under Kuh, who had led the study since 2007 and facilitated the creation of the MRC Unit of Lifelong Health and Ageing (Kuh, 2016). These tests carried an increased emotional burden for study members and changed the parameters of the science communication provided with the cards. The possibility of early diagnosis offered by the tests provided an incentive to study members to participate – ‘a bonus’, as one participant described it – but they also reasserted the duty of care the NSHD had for its participants.^49^ Should NSHD tests discover anything untoward, they were duty-bound to inform study members. This involved releasing test results to their healthcare practitioners where appropriate, but also providing study members with healthy ageing information (Kuh *et al.*, 2016: 1137). Any worries about the Hawthorn Effect had now been superseded by ethics, an acknowledgement that the study members had already been rendered an outlier community by the preceding decades. It was in this communication of diagnostic information that the linked emotional and scientific practices of the NSHD (which prevented loss to follow-up, fulfilled their duty of care, and performed kin-work) were at their most indivisible.

The 65th birthday card and parties were met with enthusiasm and garnered some press interest, but it was the 70th birthday celebrations – the ‘platinum anniversary’ – that most captured the media’s attention (McKie, 2016). This was in part due to the NSHD’s own public engagement work, which had, since the mid 2000s, deployed the birthday cards to publicise the NSHD’s work. Cards were displayed in exhibitions and at science festivals to create a timeline of researcher and participant collaboration, centring the study’s institutional identity around this relationship.^50^ During the parties, recollections were captured and videos made of study members’ experiences to populate the NSHD website and provide colour to the growing media coverage of the study. It was this coverage, and the primacy that the birthday cards took within it, that captured our interest.

**Figure 10.**
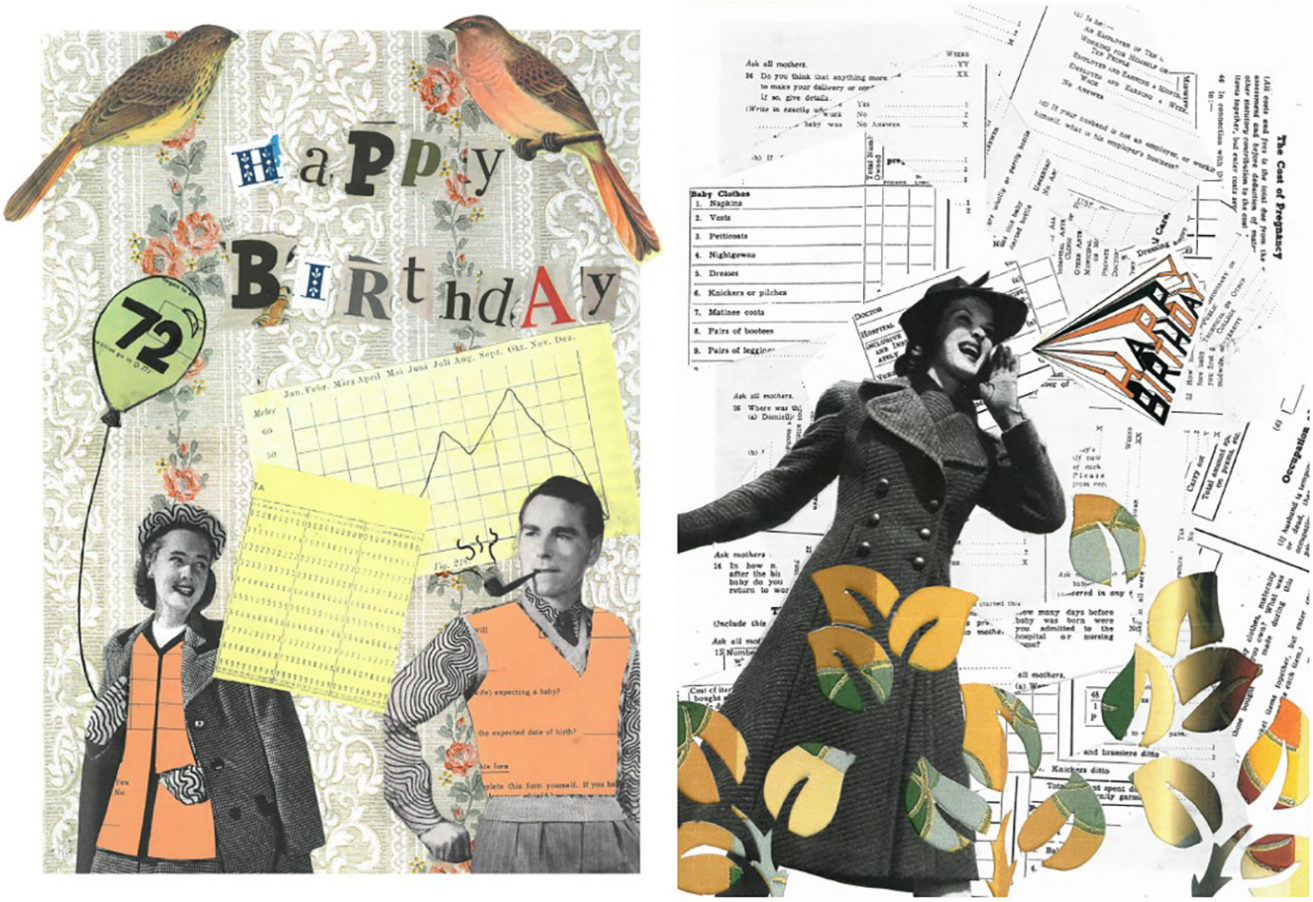
The winning 72nd birthday card images, 2018 (front and back).

We approached the NSHD with an idea for a public engagement event: a card-designing competition to be held at schools that would marry the history and science of the cohort study. The NSHD enthusiastically accepted while impressing upon us the need to produce a ‘celebratory’ and inoffensive card with participant schools. With Professor Marcus Richards and Maria Popham of the MRC Unit for Lifelong Health and Ageing at UCL, we ran a history and primary source workshop focused on the 1961 questionnaire, received by NSHD study members when they were of a similar age to the participating schoolchildren. Pupils were then tasked with designing a 72nd birthday card from archival material, previous birthday cards, and copyright-free images, using collage techniques and supervised by artist Emily Tracy (Figure 10). To ensure the schoolchildren understood the gravity of the cards they created, as thank yous and emotional objects, they were asked to imagine how it might have felt to be surveyed both as children and throughout their lives. Discussion of the 1961 questionnaire encouraged this, as did Richards and Popham leading the children through some of the physical tests performed by study members on clinic visits, such as standing on one leg with their eyes closed to check their balance. The children were also encouraged to imagine study members as ‘your grandparents’, to ‘design something they’d like’. Following the workshops, we were given access to the NSHD archives, allowed to watch researchers choose the winning card images, and later, to conduct an oral history with Kuh and Wadsworth.^51^ It was only by becoming part of the public engagement work the NSHD had already begun that we gained access to the production context of the cards discussed above, and began to grasp the evocative, intertextual, and emotional nature of the cards as objects, the apparent simplicity of the holiday snaps revealed for the complicated and involved choices they were.

In early December 2017, a large group of NSHD researchers ranked the competition entries while we watched. One was rejected because it featured a naked man’s torso, others for featuring a sunset, being ‘too busy’, or ‘too feminine for the men’. The winning images were not without controversy; some researchers feared study members might feel the NSHD was encouraging smoking by featuring the man smoking a pipe, garnering the suggestion to ‘put that naughty one on the back’ (Figure 10).^52^ While the 2018 birthday card was generally well received by study members, others correctly pointed out that the people featured on the card were dressed as if from the generation before theirs. For some, this misrepresentation was a jarring departure from cards and communications that had previously, through great effort, understood this generational cohort so well.^53^

## Conclusions

Bill Bytheway writes of birthdays that ‘through much of middle life, everyday domesticity and work dominate the course of the day. The gift-giving, cakes and singing are routine rituals that are squeezed in, reaffirming relationships in the context of relentlessly busy lives’ (Bytheway, 2011: 109). For some NSHD study members, the birthday cards acted as a similar annual affirmation of their role as participants in a study that, for the most part, occurred out of sight and out of mind as they lived their lives.^54^ On the rare occasion of a missing birthday card, the loss of reaffirmation did not go unnoticed: one participant discussing a missed card was asked, ‘“Are you a bit peeved?” “Yeh I’m a bit fed up”’.^55^ As the NSHD and its study members’ needs changed, so too did the cards that bridged their relationship. As Kuh put it, ‘The birthday card traces the changing relationship between the people who think about the study and are trying to conceive of what to do next and the people in it’.^56^ At first, the cards demonstrated the researchers’ emotional and scientific priorities, and what they felt would meet the needs of study members. Cards asked for change of address only at the moment in the study members’ lives when they were potentially leaving home. Later, as the recipients began to speak back, the cards began to reflect a more collaborative relationship, the performance of kin-work transforming into more genuine emotional communication. Cards specifically asked for study members’ views on what information they considered to be significant. Collaboratively rendered, in time they became much more than tools to prevent loss to follow-up. Instead, they became talismans for the emotional community of scholars working at the NSHD, their labour functioning as thanks and a bridge to a group that became less anthropomorphised data and more human year on year.

The study members may initially have been a group constructed according to the whims of scientific enquiry, but they nonetheless had the potential to become a textual and, later, an emotional community. Participation in the NSHD and its continued success became a shared ideology over time. We argue it was not until the birthday parties that this potential was fully realised. As survey members began to speak back, the study was seen increasingly as a collaborative endeavour. So too were the cards. The sense of shared history, obligation, and future created by membership of a textual community of shared birthday cards, newsletters, and surveys became an emotional community for *some* study members, as they attended the parties and found shared ground among their participant peers. At these parties, the sense of membership of a special group some study members felt was made a face-to-face reality; shared texts and experiences confirmed as they met one another and compared lives.

From a one-off investigation into maternity services in 1946, the NSHD is now Britain’s longest-running cohort study, the greatest ‘jewel in the crown of British science’ (Pearson, 2016b). Its significance as a case study for the history of the social sciences lies partly in this longevity. The NSHD has weathered changes in the social scientific research and funding landscape, and its study members have stayed the course. Each birthday card symbolises another year as the longest-running cohort study; of scientific data collection; of commitment from researchers and study members; another year of feelings felt. It is only by taking the birthday cards seriously – not just as institutional objects, but as emotional objects – that we were able to trace the development of the NSHD’s institutional identity and the transition of its study members from cohort to community. Only through attending to the feelings of researchers and study members, and participating in the NSHD’s public engagement and science communication work, were we afforded insight into processes that have ensured the study’s success and that would otherwise be hidden. As researchers, we call on historians of science and social science to look for the feelings that thread throughout these fields, to excavate the emotions that entangle their subjects, and to reveal further hidden histories.

## Notes

This article uses images, documents, and interviews held in the NSHD archive which was uncatalogued at the time of publication. Throughout the article every effort has been made to give as full a citation as the material and confidentiality allows. The images reproduced here are done so with the kind permission of the NSHD. All images remain the property of the NSHD. We are grateful to the staff at the NSHD for the use of their materials. We are especially grateful to Diana Kuh and Michael Wadsworth for allowing us to observe the card choosing process and recording an oral history, and to Maria Popham for aiding our use of the then uncatalogued NSHD archive. Earlier versions of this paper were read by Placing the Public in Public Health team at the London School of Hygiene and Tropical Medicine. Our thanks go to the whole team, but especially to Alex Mold, for her patience, helpful comments and suggestions.

1. NSHD participant, ‘NSHD Interview 20’, 2010, interviewed by Sam Parsons, p. 67; NSHD participant, ‘NSHD Interview 23’, 2010, interviewed by Sam Parsons, p. 71.

2. A cohort study is the study of a group of individuals who are linked in some way, or who have experienced the same significant life event within a given period, such as birth. Cohort studies include measures of characteristics at two or more points in time. The studies that followed the NSHD (1946) are: the National Child Development Study (1958), the 1970 British Cohort Study, the Avon Longitudinal Study of Parents and Children (1991), and the Millennium Cohort Study (2000). Each study follows a different generation, which allows for comparison between the cohorts.

3. NSHD questionnaire, 2015.

4.
*Study members* is the preferred term of the NSHD for its participants. All cards (except 1973, which is missing) may be viewed at ‘Birthday Card Gallery’, *MRC National Survey of Health and Development*, available at: https://www.nshd.mrc.ac.uk/archive/birthday-card-gallery/.

5. The term *textual community* is drawn from Brian Stock’s work on medieval religious communities, where he uses the term to describe the social circles that are formed by sharing texts and consequently the shared knowledge, expectations, and rules of behaviour based in the texts’ ideology. The term *emotional community*, drawn from Barbara Rosenwein’s work, describes a similar concept, though, as will later be discussed, it concentrates more specifically on shared emotional regimes and relationships rather than the texts that foster them (Grisé, 2002; Rosenwein, 2002; Stock, 1983).

6. While Mass Observation’s reach was comparatively small (with 500 diarists and 1095 people regularly completing directives during the Second World War), the organisation published over 20 books between 1937 and 1950, and staff members produced numerous reports for newspapers and broadcast media (Summerfield, 1985).

7. NSHD questionnaire, 1946.

8. NSHD questionnaire, 1948.

9. NSHD questionnaire, 1950.

10. NSHD questionnaire, 1957.

11. NSHD questionnaire, 1961.

12. NSHD 16th birthday card, 1962.

13. NSHD questionnaire, 1966.

14. NSHD participant, ‘NSHD Interview (May)’, 2010, interviewed by Fiona Rait, p. 57; NSHD participant, ‘NSHD Interview (23 June)’, 2010, interviewed by Sam Parsons, p. 63.

15. This feeling was not unique to NSHD study members: Mass Observation correspondents in the 1990s felt similarly about the archivists and researchers (Shaw, 1994: 1399; Sheridan, Street, and Bloome, 2000: 70).

16. D. Kuh and M. Wadsworth (11 January), 2018, Interview by Authors.

17. The worth instilled by anniversaries has been similarly deployed by those wishing to bolster the National Health Service, the very enduring nature of the NHS becoming part of the arguments for its preservation (Crane, 2020).

18. ‘NSHD Interview (23 June)’, p. 48; ‘NSHD Interview (May)’, p. 55.

19. Kuh and Wadsworth, interview.

20. NSHD questionnaire, 1961.

21. NSHD questionnaire, 1968.

22. Kuh and Wadsworth, interview.

23. NSHD 21st birthday card, 1967.

24. NSHD questionnaires, 1966, 1968, 1969, 1971; NSHD 21st birthday card.

25. NSHD 21st birthday card.

26. Kuh and Wadsworth, interview.

27. NSHD 22nd birthday card, 1968.

28. NSHD questionnaires, 1977, 1972.

29. Kuh and Wadsworth, interview.

30. NSHD questionnaire, 1982.

31. NSHD questionnaire, 1982.

32. ‘NSHD Interview 23’, pp. 70–2.

33. Kuh and Wadsworth, interview.

34. NSHD participant, ‘NSHD Interview 30’, 2010, interviewed by Sam Parsons, p. 46.

35. Kuh and Wadsworth, interview.

36. NSHD card-packing procedure, 1991, p. 2; NSHD card-packing procedure, 1992, p. 2.

37. Card Packing Procedure, 1992: 1.

38. Kuh and Wadsworth, interview.

39. NSHD birthday card, 1989.

40. Kuh and Wadsworth, interview.

41. Kuh and Wadsworth, interview.

42. 21st Birthday Card, 1967.

43. NSHD newsletter, 2003.

44. NSHD questionnaire, 2006.

45. National Survey of Health and Development News’ (2012) NSHD.

46. Kuh and Wadsworth, interview

47. Kuh and Wadsworth, interview

48. NSHD birthday card, 1997.

49. NSHD participant, ‘NSHD Interview 28’, 2010, interviewed by Sam Parsons, p. 113.

50. M. Popham, ‘2018 Birthday Card: Responses From Study Members’, correspondence with authors, 2018; NSHD, ‘Birthday Card Gallery’, https://www.nshd.mrc.ac.uk/archive/birthday-card-gallery/

51. Kuh and Wadsworth, interview.

52. Elizabeth and Payling (2017) ‘Observational Notes From 2018 NSHD Card Choosing’.

53. Popham, ‘2018 Birthday Card’.

54. NSHD participant, ‘NSHD Interview 11’, 2010, interviewed by Fiona Rait.

55. NSHD participant, ‘NSHD Interview 24’, 2010, interviewed by Sam Parsons, p. 115.

56. Kuh and Wadsworth, interview.
